# Nursing students motivation toward their studies – a survey study

**DOI:** 10.1186/1472-6955-7-6

**Published:** 2008-04-25

**Authors:** Kerstin EL Nilsson, Margareta I Warrén Stomberg

**Affiliations:** 1 School of Life Sciences, University of Skövde, Skövde, Sweden; 2 Institute of Health and Care Sciences, The Sahlgrenska Academy at Göteborg University, Göteborg, Sweden

## Abstract

**Background:**

This study focuses on Swedish nursing students' motivation toward their studies during their three year academic studies. Earlier studies show the importance of motivation for study commitment and result. The aim was to analyze nursing students' estimation of their degree of motivation during different semester during their education and to identify reasons for the degree of motivation.

**Methods:**

A questionnaire asking for scoring motivation and what influenced the degree of motivation was distributed to students enrolled in a nursing programme. 315 students who studied at different semesters participated. Analyzes were made by statistical calculation and content analysis.

**Results:**

The mean motivation score over all semesters was 6.3 (ranked between 0–10) and differed significantly during the semesters with a tendency to lower score during the 5th semester. Students (73/315) with motivation score <4 reported explanations such as negative opinion about the organisation of the programme, attitude towards the studies, life situation and degree of difficulty/demand on studies. Students (234/315) with motivation score >6 reported positive opinions to becoming a nurse (125/234), organization of the programme and attitude to the studies. The mean score value for the motivation ranking differed significantly between male (5.8) and female (6.8) students.

**Conclusion:**

Conclusions to be drawn are that nursing students mainly grade their motivation positive distributed different throughout their entire education. The main motivation factor was becoming a nurse. This study result highlights the need of understanding the students' situation and their need of tutorial support.

## Background

The nursing students' motivation towards their studies is a question of energy, such as processes starting, sustaining and directing their study behavior. This paper focuses on Swedish nursing students' own assessment of their motivation towards their studies during their three year academic education. Notable is that the students' interest and commitment vary during their education. It is therefore interesting to systematically study the students' own experience of how motivated they felt.

Motivation can be seen either as an intrinsic or an extrinsic factor. Enjoying learning for its own sake or positive feedback on learning outcomes are examples of intrinsic motivation. Accordingly there is a built-in pleasure for the activity itself. Intrinsically motivated students have a driving force to learn, perform, and a wish to succeed. Attaining consequences (for example a desired grade) or avoiding punishment (for example from parents) outside oneself briefly explains extrinsic motivation. Extrinsically motivated student will perform for attaining a desired grade or some other external reward [1].

Intrinsic motivation has been found to be an important factor in children's education [2]. There are strong degrees of continuity in individuals' academic intrinsic motivation, during education in elementary and high school years [3]. According to Gottfried and her colleagues this academic intrinsic motivation will not so easily be changed in adolescence.

In a study of self-efficacy, intrinsic and extrinsic motivations as predictors for students' engage or not in academic work were found that self-efficacy and intrinsic motivation were correlated to academic identification, and were predictors to meaningful cognitive engagement. Furthermore extrinsic motivation was found to be a predictor to shallow cognitive engagement in learning tasks [4].

Another important theory about motivation is goal theory, which was initially divided in two achievement goals. The first one, mastery goals, focused on development of competence and tasks and is related to learning outcomes. The second one, performance goals, dealt with relative competence in relation to others and is a more self-centered goal [5]. In longitudinal studies it was shown that active learning goals were more likely to sustain motivation than performance goals, ability-linked goals and normative goals and that outcome goals (wanting a good grade) were equally related to learning goals and ability goals in contrast to performance avoidance goals (avoidance of failure) [6]. Students enrolled in master level studies were found to have high achieving tendencies and academic abilities in their first semester, which support achievement motivation theory [7]. Learning outcomes and self-centered goals are not enough bases for study motivation, the value of the acquired competence in the future should be added as a motivation factor [8]. In a study of motivation among first-year nursing students, goals and the future time perspective theories were combined. This study found that the students could be motivated by the present studies leading to the future utilities as registered nurses and that both the present and the future might be regulated internally or externally. These four dimensions of a goal have different influence on motivation. Internally regulated students were more task-oriented and more interested in the course and performed well. Externally regulated students used more avoidance ego goals, were less excited and performed worse. Students, who also find the courses useful for the future, not only for the training, were more excited, more motivated for their study and reached better result than did the students who found the courses just relevant for training. Type of utility did not effect approach and avoidance ego goals [9].

It was found that nursing students were motivated by the desire to help others and to do something useful. Despite the fact that nearly half of them did not choose nursing studies as there first choice [10]. Caring for others was found to be a main motivator for female nursing students choosing nursing education, but power and empowerment of self and others are the dominating factors for their choice [11]. Male nursing students' choice of nursing education depends on the fact that they consider that the nursing profession offers job security, opportunity and flexibility [12] as well as the desire to care for others [13].

The road to a bachelor's degree in nursing might be filled with both possibilities and obstacles. A study found that eight out of 76 students did not complete their studies despite the fact that they had been motivated towards nursing studies. Besides personal reasons such as sickness; the students dropped out on account of study results. Missed examinations in theoretical as well as in practical subjects reduced the motivation to make efforts in continuing their nursing studies [14]. Similar results, such as academic difficulties, wrong career choice and personal and social problems, were found in a study where exit-interviews were accomplished when nursing students terminate their nursing education ahead of time [15].

How nursing students who complete their nursing education are motivated towards their studies during the entire program is not studied, particularly not in a Swedish context. One can presume that the individual student's interest and motivation towards their studies vary during their education and thereby need different support from tutorials and teachers in different phases of their studies. Therefore, it is of importance to study the students' self rated motivation and explanations to the degree of motivation during their three year nursing education.

The aim of the study was to analyze how nursing students estimate their degree of motivation at different semesters during their education and identify reasons for the motivated grade.

## Methods

The study was performed during April-May 2006. Data were collected by both of the researchers. During this time one of the researchers at random attended a voluntary lecture in a nursing program at a medium-sized university in mid-Sweden to give information about this study. After informed consent from the students; a questionnaire was distributed to the students who were present and willing to participate in the study. The main question was to self-grade question about their own assessment of their motivation on a scale graded from 0 to 10, were 0 was not motivated at all and 10 was highly motivated. This rating scale has labeled end points, which specify the opposite extremes of a continuum. A rating scale with odd numbers is recommended to allow for a neutral midpoint, which is why 11 points was chosen in this study [16]. Thereafter followed an open-ended question asking which factors exerted an influence on their motivation. The researchers distributed and collected the completed questionnaires during the same lecture. The data collection was carried out during a short period with similar circumstances.

### Participants

Students being enrolled in the nursing program at the University were offered to participate in the study. The nursing program runs for 3 years, six semesters. Only students who attended the random lecture were offered to participate. In each semester 100 students were enrolled and in mean 53% participated in the actual lectures. This figure (53%) is a common figure for participation in Swedish university lectures as participation is not obligatory. None of the students invited to participate in the study declined. The questionnaire was given once to students at the first; second, third, fourth, fifth and sixth semester.

### Statistics and Analysis

All data were stored in a computerized database and processed using the Statistical Package for the Social Sciences (SPSS version 14.0). Data were sorted into the same areas as asked for in the questionnaires. The statistical calculations included frequency counts, percentages, mean value and standard deviation. To compare the graded motivation between the semesters the non parametric Kruskal-Wallis test was used. Mantel's test was applied for the over all comparison between sexes and motivation with the semester as a background variable. Data were further analyzed according to a gender perspective and a motivating score value <4 and >6 as well as the extreme values of a 0 or 10 score. The reliability of this instrument accurate reflects the true score of the attribute investigated [16].

The open-ended questions in the questionnaire were inductively analyzed in a systematic way that lead to the drawing of inferences [16]. The process used when analyzing the data was similar to content analysis. The characteristic of this method is the systematic distillation through analysis of verbal or written data in order to describe and quantify specific phenomena into fewer content-related categories sharing the same meaning. The analysis ends with the answers on; "What occurs and how often does it happen?" [17]. The following sentence 'I am uncertain if I have all that knowledge to be requested in the profession' illustrates an answer in the category 'The certificate and the profession frightened the student'.

Categories occurred in the data are summarized in Table 1. A category can accordingly be measured as a positive valued category as well as a negative valued category. Each individual could answer more than one reason for their graded motivation score. This inductive analysis of the individuals' explanation of motivation has been linked to her or his degree of motivation. To avoid influence of the researchers' pre-conceptions of the issue in question, awareness of this risk followed the research process. During analysing phase the analysis were performed separately by the two researchers and thereafter compared. The level of agreement between the two co-examiners was about 95% and points of disagreement were resolved through discussion.

**Table 1:** Categories emerged from the analyzed data

**Categories emerged**	**Positive valued**	**Negative valued**
The degree of difficulty/demand of the studies	The degree of difficulty/demand was experienced as stimulating	The degree of difficulty/demand was experienced as too heavy
The teachers engagement	Very engaged teachers	Not engaged teachers
The organisation of the program	A stimulating organisation of the program was noticed	No stimulating organisation of the program was noticed
Contents in the studies associated to the profession	The certificate to be a nurse is attractive – long for working as a nurse	The certificate and the profession frightened the student
The attitude towards the studies	Having a positive attitude towards the studies	Having a negative attitude towards the studies
The study result achieved	Achieving a good study result	Not achieving a good study results
The life situation surrounding the studies	Having a good life situation	Having a bad life situation
Friendship during the studies	A good friendship is experienced	A bad or no friendship is experienced

### Ethics

Approval for this study was provided by the Head of the Institute at the University, the responsible person for quality assurance at the University and the students' association. Both of the researchers are employed at the University. Together with data collection the students were informed and gave their written informed consent.

## Results

Of totally 597 possible participating students 315 were present at the randomly chosen/visited lectures. All students present were offered to participate and all of them accepted to participate, accordingly the response rate was 100%. However, the response rate in relation to the whole student population was thereby 53% distributed to 18% male students and 82% female students. The students mean age was 27 yrs (a normal mean age for Swedish nursing students) the 1^st^, 2^nd ^and 3^rd ^semester, 29 yrs 4^th ^semester, 28 yrs 5^th ^semester and 32 yrs the 6^th ^semester. The mean motivation score over all semesters was 6.3.

**Figure 1:**
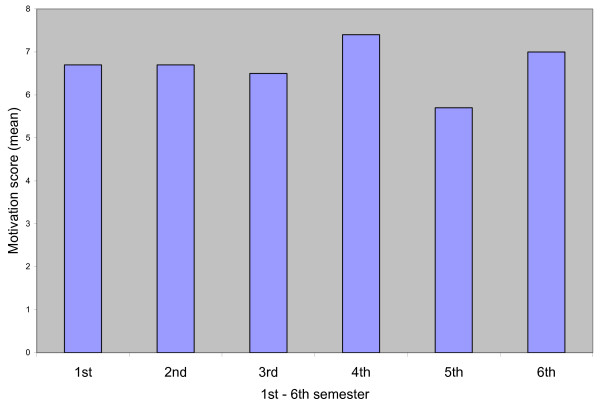
The mean motivation score measured in the 1^st ^– 6^th ^term.

The motivation score for each semester is shown in Figure 1. The mean score differed significantly between semesters. The standard deviation for the score value varied from 1.8 – 2.3. The gradation of the students' motivation was not similar during the 6 semesters (p 0,006). During the 1^st^, 2^nd ^and 3^rd ^semester the mean was 6.7; 6.7; 6.5 respectively, (fig 1) and during the 4^th^, 5^th ^and 6^th ^semester it was 7.4, 5.7; 7.0 respectively (fig 1).

When analyzing the explanation from all the students' scoring of the 0–10 scale, the data were analyzed divided into to two parts; motivation score ≤4 and motivation score ≥6 defined as low and high motivation respectively and this is shown in table 2, 3. In table 2 the statements are outlined when scoring the motivation ≤4.

**Table 2:** Motivation score ≤4, defined as low motivation and the explanations/statements

**Categories emerged as explanations for low motivation**	**Frequencies of statements in data**
The degree of difficulty/demand on the studies was experienced as too heavy	13
Not engaged teachers	6
No stimulating organisation of the program was noticed	14
The certificate and the profession frightened the student	9
Having a negative attitude to the studies	14
Not achieving a good study results	2
Having a bad life situation	14
A bad or no friendship to fellow-students was experienced	1

Frequencies of responses scored ≤4	73

**Table 3:** Motivation score ≥6, defined as high motivation and the explanations/statements

**Categories emerged as explanation for high motivation**	**Frequencies of statements in data**
The degree of difficulty/demand on studies was experienced as stimulating	5
Very engaged teachers	18
A stimulating organisation of the program was noticed	29
The certificate to be a nurse is attractive – long for working as a nurse	125
Having a positive attitude to the studies	29
Achieving a good study result	11
Having a good life situation	6
A good friendship to fellow-students was experienced	11

Frequencies of responses scored ≥6	234

Statements made when justifying low motivation were in most cases; the organization of the program is not stimulating, a negative attitude to the studies, having a bad life situation and an experience of too heavy degree of difficulty/demand on studies.

The outstanding statement for a high motivation score was the students' wish to become a nurse and obtain their degree as registered nurse. At the same time several students found the organization of the program stimulating and they had a positive attitude to the studies. Significantly more explanations were used to a high motivation score (≥6) compared to explanations for a low motivation score (≤4), n 234 and n 73 statements respectively.

The minimum score value differed from 0–4 during the various semesters while the maximum score value was 10 in all semesters when analyzing the whole data.

Grading the motivation score to a maximum of 10 was done by totally 28 out of 315 students. The main reasons for that were the students' longing for working in the nursing profession followed by students' positive attitude to their studies. The lowest grading score, 0 was given by one student. The explanation given was that the choice of the nursing profession was wrong for the student. This student, who had carried out the 5^th ^semester, had not achieved acceptable study results (when checking the amount of credits passed).

The mean score value for the motivation ranking was 5.8 for men and 6.8 for women. When using Mantel's test with semester as the background variable we found significant differences, p 0,0007 between sexes where female were more motivated. Statements mostly given as reason for men's positive motivation score was the students longing for working in the nursing profession, the organization of the program was stimulating and their own study results were good.

When analyzing the importance of a good friendship to fellow-students separately it was given as a positive explanation in 15 cases, where 7 explanations was found in the 2^nd ^semester, n3 in 3^rd ^and 5^th ^semester, n1 in the 1^st ^and 4^th ^semester and no one in the last semester.

## Discussion

The aim of the study was to analyze the nursing students' degree of motivation at different semesters during their education and identify reasons for the motivated grade. To be noted in this study was that the students graded their motivation quite similar during the 1^st^, 2^nd ^and 3^rd ^semester (mean value 6.7; 6.7; 6.5 respectively, see fig 1). There was a small difference during the 4^th^, 5^th ^and 6^th ^semester (mean value 7.4; 5.7; 7.0 respectively, see fig 1). In accordance to McEvan et al, [7] and Simons et al, [8] it would be expected that the motivation should be graded more high for the first and the last semester just when entering the studies and just before entering employment. However, this was not found in this study. The relatively high score in semester four might be explained by the content in the courses in this actual nursing school focusing on clinical skills compared to other semesters, which are more theoretical. When training in this semester the students most likely get a new insight to their future occupation as registered nurses. This laboratory clinical training situation was found stimulating and could be compared to vital learning experience in the clinical area [18]. The relatively low graded score for semester five could probably be explained by theoretical content in semester five contrasting semester four simultaneously to a consciousness of the future professional demand. It might also be a result of tiredness of theoretical studying situation and longing to become a registered nurse. Furthermore discussions concerning the final examination thesis had occurred at the university; voices had been raised towards the examination, which might have influenced some students and reduced their motivation. In the last clinical courses they finally have to bring together their clinical and theoretical ability necessary to become a registered nurse. This explanation could be seen in the light of external motivation theory [1] and goal motivation theory [5] as the students seem to look forward and long for their employment.

The study result showed that extrinsic motivators, such as teachers' involvement, organization of the program, contents in the studies associated to the profession, life situation and friendship during the studies were more commonly used as explanation to the motivation score than was intrinsic factors such as attitude to the study and study result. This is also similar to Newby's findings, showing that extrinsic motivators are more frequent referred to than intrinsic motivators [19]. In connection to this finding one question can be raised: Do the educators meet the students' learning interests. The use of writing portfolio and its assessment as well as justification of the students' learning process enhance the students' learning outcomes [20]. A portfolio could be one way to match the students learning interests.

The study result found that nine students were frightened to be employed registered nurses. The explanation to that could be similar to others who found that motivation is effected substantially by self-efficacy or students' beliefs regarding their ability to meet professional demands [4,21].

It is also interesting to note that the overall mean motivation score was higher for female students than for male students. There is no obvious explanation for this. Jacobsson [22] found that female students had higher intrinsic motivation than male and that female students exert themselves more as well as having a more thought-out strategy for their studies. This might be an explanation for the gender differences in our findings. The nursing profession is found to be a typical female profession. In Sweden 91 percent of the gainfully employed registered nurses are female [23]. According to Glossop [15] the male nursing students low motivation score might have been influenced by wrong career choice.

When analyzing the students' explanation for a high motivation score (≥6) compared to explanations for a low motivation score (≤4) it was interesting to find more frequent explanations for a high motivation score compared to a lower score. The fact that the students were the subject of this study might influence there attitude in a positive direction giving more positive statements in the open-ended questions. An interpretation of Bandura [21] implies that students' possibility to perceive their own competences and ability is a positive impact motivation factor.

Another explanation might be that students with high motivation scores probably also have a higher level of self-efficacy and therefore easier find positive explanations and achieve good study results. Kloster [14] found missed examinations reduced motivation scores. It would have been interesting to find out if that explanation was able to apply also in this study, but there was not enough foundation. The only student in this study that scored 0 as motivation grade had failed in several examinations, which correspond to Klosters findings regarding reasons for reducing a student's motivation. To speculate about other reasons to fewer explanations concerning low motivations it might be easier to talk about positive arguments than negative ones. However, the result showed that one main explanation to a low motivation score was when the student had a bad life-situation such as a stressful situation at home. In this result the importance of the life-situation was distinctly emerged as an explanation to low motivation. This result indicates the importance of giving the students tutorial support in a broader perspective than just academic achievement, for example by promoting flexibility in planning personal study programmes [24]. This also emphasizes the teachers' and the University's responsibility to support the students during their education especially when their motivation for the studies are weakening; as motivation has great impact on learning outcome. On the other hand the study did not show that a good life-situation seemed to influence a high motivation sore.

Using a rating scale might be seen as a limitation as it just grades the students' opinions about their motivation. Another type of motivation instrument would have measured their motivation in more detail. The limitation of comparing students from deferens semesters was compensated by students attaining the same curriculum, studying at the same university, and no organisational changes were done during the study period.

## Conclusion

Conclusions to be drawn are that nursing students mainly grade their motivation positive and similar distributed throughout the entire education. The main motivation factor was extrinsic and goal oriented; namely becoming a nurse. The nursing students mentioned intrinsic motivation factors as explanation for their degree of motivation. This study result highlights the need of understanding the students' situation and their need of tutorial support. Furthermore the organizations of the program need to be developed in co-operation with the students to match content in courses and the students' different degree of motivation during their academic years. In further studies we will follow the degree of motivation in a cohort of students (one class) during their entire three years of study.

## Competing interests

The authors declare that they have no competing interests.

## Authors' contributions

KELN: Study design, data collection, data analysis, writing the manuscript. MIWS: Study design, data collection, data analysis, writing the manuscript. All authors read and approved the final manuscript.

## Pre-publication history

The pre-publication history for this paper can be accessed here:


